# Ecohydrological Responses to Diversion of Groundwater: Case Study of a Deep-Rock Repository for Spent Nuclear Fuel in Sweden

**DOI:** 10.1007/s13280-013-0404-8

**Published:** 2013-04-26

**Authors:** Kent Werner, Per Collinder, Sten Berglund, Erik Mårtensson

**Affiliations:** 1 EmpTec, Larmvägen 8, Täby, 187 75 Sweden; 2 Ekologigruppen AB, Åsögatan 121, Stockholm, 116 24 Sweden; 3 HydroResearch AB, Stora Marknadsvägen 15S, 12th Floor, Täby, 183 34 Sweden; 4 DHI Sverige AB, Kyrkogatan 3, Lund, 222 22 Sweden

**Keywords:** Tunneling, Rock cavern, Hydrological modeling, Environmental impact assessment, Groundwater-dependent ecosystems, Forsmark

## Abstract

Planning and license applications concerning groundwater diversion in areas containing water-dependent or water-favored habitats must take into account both hydrological effects and associated ecological consequences. There is at present no established methodology to assess such ecohydrological responses. Thus, this paper describes a new stepwise methodology to assess ecohydrological responses to groundwater diversion from, e.g., water-drained pits, shafts, tunnels, and caverns in rock below the groundwater table. The methodology is illustrated using the planned deep-rock repository for spent nuclear fuel at Forsmark in central Sweden as a case study, offering access to a unique hydrological and ecological dataset. The case study demonstrates that results of ecohydrological assessments can provide useful inputs to planning of monitoring programs and mitigation measures in infrastructure projects. As a result of the assessment, artificial water supply to wetlands is planned in order to preserve biological diversity, nature values, and vulnerable species.

**Electronic supplementary material:**

The online version of this article (doi:10.1007/s13280-013-0404-8) contains supplementary material, which is available to authorized users.

## Introduction

Environmental impact assessments (EIA) are commonly performed and reported as part of planning and license applications for infrastructure projects. These EIA must identify and assess risks, associated with various types of disturbances during construction and operation, to humans and the surrounding environment. Examples of typical disturbances in infrastructure projects (that include pits, shafts, tunnels, caverns, or other types of cavities in rock) are landscape encroachments, noise, vibrations, and dusting due to rock blasting and crushing, as well as emissions of water and air pollutants from the use of contract machines.

If the cavity is located below the groundwater table, inflowing groundwater must be diverted in order to keep the cavity drained. In addition to the disturbances mentioned above, in such cases the EIA must also include an assessment of the resulting groundwater-table drawdown and other hydrological effects in the surroundings of the cavity. Moreover, depending on the characteristics of the area in which the cavity is located, it is required that the EIA consider consequences for house foundations and infrastructure, water-supply wells, agriculture, forestry, and other ecological conditions. In particular, planning and license applications for construction and operation of water-drained cavities in areas that contain natural habitats for water-dependent or water-favored flora and fauna need to take into account ecohydrological responses to the diversion of groundwater, i.e., hydrological effects and associated ecological consequences.

Compared with other environmental disturbances, such as noise and vibrations, the hydrological effects of groundwater diversion from cavities in rock and associated ecological consequences are relatively difficult to predict, and any such predictions are normally subject to uncertainty. For instance, detailed predictions of hydrological effects may require extensive data on geometries and hydraulic properties of fracture zones in the rock as well as the hydraulic properties of the interface between regolith and rock (e.g., Olofsson 1991, 1994).

In Sweden, there are many theoretical and practical studies concerning groundwater inflow to drained cavities below the groundwater table and associated hydrological effects (Olofsson 1991; Cesano 2001). Studies related to ground subsidence and other geotechnical problems, caused by groundwater diversion from rock tunnels in urban areas, were initiated in Sweden in the 1960–1970s (Broms et al. 1976; Knutsson and Morfeldt 2002). During the last few decades, much of the research on constructions in rock has been focused on issues such as grouting, secondary hydrochemical effects of groundwater diversion, and other types of interactions between cavities and the surrounding rock (Eriksson 2002; Gustafson 2009; Mossmark 2010). Practical experience of ecohydrological responses reported in the literature concerns, for example, ecological consequences of surface-water drainage (Simonsson 1987) and disturbances from tunneling projects on forestry, agriculture, and water-dependent or water-favored habitats (Tunnelkommissionen 1998; Florgård et al. 2000; Karlsrud et al. 2003).

Assessments of ecohydrological responses to groundwater diversion require different types of data, modeling tools, and a methodology to utilize the data in the tools. The integrated subject *ecohydrology* (or *hydroecology*) is relatively new and it has been given increasing attention during recent years among the ecological and hydrologic scientific communities, including debates regarding the subject’s scope and definition (Baird and Wilby 1999; Zalewski 2000; Nuttle 2002; Bonell 2002; Bond 2003; Hanna et al. 2004; Eamus and Froend 2006; Gasca and Ross 2009; Hancock et al. 2009; Bertrand et al. 2012). At present, the subject is rather immature and it contains many open, basic research questions. As a result, there is as yet no established methodology on how to assess ecohydrological responses to groundwater diversion in areas with water-dependent or water-favored habitats. This paper aims to contribute to the development of the subject and its applications, by suggesting a methodology and providing practical examples.

Using data and modeling results from the Forsmark site in Sweden as case study, the paper describes a stepwise ecohydrological-response assessment (ERA) methodology that links diversion of groundwater from the rock to hydrological effects and ecological consequences in surface systems. Forsmark has been selected as the site for the planned Swedish deep-rock repository for spent nuclear fuel, and it offers access to a unique hydrological and ecological dataset (Kautsky et al. 2013). The construction, operation, and decommissioning of the repository require assessments of various types of risks to human health and the environment. These assessments concern risks on a wide range of spatial and temporal scales. Long-term risks, on the time scale of 100 000 years, are associated with potential post-decommissioning radionuclide releases and subsequent transport towards and within the biosphere (SKB 2011a; Avila et al. 2013; Kautsky et al. 2013). The assessments of long-term risks include other applications of ecohydrological methods than those presented in this paper. Specifically, conceptual and quantitative models of radionuclide transport within the biosphere are based on integrated descriptions of hydrological and ecological processes (Berglund et al. 2009). The present study concerns environmental impacts, other than radionuclide releases and transport, during the construction and operation phases of the repository. The total time scale of these phases is obviously short, as compared to other time scales related to disposal of radioactive waste.

## Materials and Methods

### The Planned Deep-Rock Repository at Forsmark

Forsmark is located on the coast of the Baltic Sea in central Sweden. For an overview map of Sweden showing the location of Forsmark, see Kautsky et al. (2013). The so-called KBS-3 method for disposal of spent nuclear fuel in Sweden (SKBF/KBS 1983) implies that copper canisters with a cast-iron insert containing the spent fuel are enclosed by bentonite clay and deposited at a depth of approximately 500 m in crystalline rock. The subsurface part of the planned repository at Forsmark (Fig. S1, in Electronic Supplementary Material) consists of a spiral-shaped access ramp and vertical shafts, a central area with a number of rock caverns, and a repository area with tunnels for deposition of canisters (SKB 2009). The total tunnel length will be ca. 70 km, and the construction, operation, and decommissioning phases will comprise a total time period of 60–70 years. During the construction and operation phases the access ramp, the central area, and the shafts will be open. During repository operation, different deposition tunnels in the repository area will successively be constructed, used for canister deposition and thereafter backfilled. Hence, all deposition tunnels will not be open simultaneously at any point.

The drainage water that will be pumped away during construction and operation of the repository will be a mixture of inflowing groundwater and process water, that is, water used during drilling and for cleaning of rock walls and construction equipment. Prior to pumping to the ground surface, the drainage water will be treated by means of sedimentation and oil separation in one of the rock caverns in the central area. Subsequent to further polishing steps at the ground surface, drainage water will be discharged to the Baltic Sea (SKB 2009).

### Overview of the Forsmark Site

Extensive, multidisciplinary site investigations, processing of emerging data, and site-descriptive modeling were performed during the period 2002–2008 (Lindborg 2008; SKB 2008). The overall objectives of the site investigations and the modeling have been to develop and document an integrated description of the site, serving as a basis for studies of the repository layout (SKB 2009) as well as assessments of long-term radiological safety (SKB 2011a) and environmental impacts during construction and operation (SKB 2011b).

Forsmark contains many water-dependent or water-favored habitats that potentially could be affected by groundwater diversion during construction and operation of the repository. Examples of such habitats include small wetlands, in the form of fens and ponds, and coniferous forests located on lime-rich soil. The wetlands and forests host species that are considered to be worthy of protection. Red-listed wetland species (Gärdenfors 2010) include pool frog (*Rana lessonae*) and fen orchid (*Liparis loeselii*). These two species are also legally protected according to the EU’s Habitats Directive (European Commission 1992), which in Sweden is incorporated into the Species Protection Ordinance (Miljödepartementet 2007). The high nature values of Forsmark are the result of the site’s near-coastal location, the flat topography, a fast shoreline displacement, and the small but important topographic variations. Other important factors include the lime-rich soil and Forsmark’s relatively undisturbed location.

The regolith (unconsolidated deposits overlying the rock) in Forsmark is dominated by relatively permeable till. The groundwater table is shallow and generally follows the ground-surface topography, which causes small-scale groundwater flow systems near the ground surface to overlie larger-scale flow systems in the rock. The upper 100–150 m of the granitic rock (Fig. S2 in Electronic Supplementary Material) contains a well-connected network of geological structures, consisting of so-called sheet joints, i.e., geological structures that may have high horizontal permeability, and steep fracture zones that locally are in contact with the regolith (Follin 2008).

### Ecological Field Inventories and Classification of Nature Values

The first step of the ERA methodology includes field inventories for geographical delineation and classification of water-dependent or water-favored habitats, hereafter called *nature objects*. At Forsmark, preparatory work for delineation and classification included studies of aerial photographs and maps of, for example, land use and vegetation coverage. Photographs and maps were used to identify moist and wet areas having prerequisites for hosting rare, water-dependent, or water-favored nature types and species. According to established EIA practice in Sweden, the system shown in Table 1 was used for classification of nature values at Forsmark.

**Table 1:** Classification schemes for nature values, hydrological sensitivities, hydrological effects, and ecological consequences. *NA* not applicable, *GTD* groundwater-table drawdown, *IAB* influence-area boundary (annual average GTD = 0.1 m)

Type of classification	Class 1	Class 2	Class 3	Class 4	Class 5	Class 6
Nature values	National	Regional	Local	Municipal	NA	NA
Hydrological sensitivities	Very high	High	Sensitive	Less sensitive	Not sensitive	NA
Hydrological effects	GTD > 2 m	GTD = 0.5–2 m	GTD = 0.1–0.5 m	IAB = 0–100 m	IAB = 100–200 m	IAB = 200–300 m
Ecological consequences	Very large	Large	Noticeable	Small	Very small	No conseq.

Object-specific classifications take into account factors such as rarity of nature types, including the presence of Natura 2000 nature types according to the EU framework, and rarity of species, taking into account occurrences of red-listed and/or legally protected species. Moreover, the classification methodology considers size and spatial continuity of delineated nature objects, and the presence of ecologically important structures or functions. It should be emphasized that nature-value classifications require many years of practical experience, as well as sample controls and other forms of quality checks.

Comprehensive ecological field inventories were performed at Forsmark in an investigation area covering ca. 10 km^2^. Rich (alkaline) fens were investigated using the national method for rich-fen inventory in Sweden (Sundberg 2007). The inventories were focused on amphibians, plants and the so-called indicator species, i.e., specific mosses that are typical of rich fens. Forest inventories were performed using a method developed by the Swedish Forest Agency (Skogsstyrelsen 2005) for inventories of key habitats. Specific inventories were performed of ground- and wood-living fungi, land mollusks, dragonflies, and bottom fauna, aquatic vegetation, and fish in lakes.

### Classification of Hydrological Sensitivity

The second step of the ERA methodology involves classification of delineated nature objects according to their sensitivity to changes in the hydrological conditions. The objective of this classification is to define object-specific threshold values for changes in groundwater-table depths or surface-water depths for nature-type transitions and losses of nature values. The hydrological sensitivity is related to water-supply mechanisms and water balances, which in turn are governed by topographical, meteorological, hydrological, and other factors that collectively contribute to the ecological status of an object. Hence, the hydrological sensitivity can be viewed as a combined measure of the intrinsic sensitivity, governed by nature type and species, and the hydrological vulnerability, which is governed by factors such as water-supply mechanisms and presence of low-permeability layers. For instance, the methodology for classification of hydrological sensitivity takes into account that an object’s vulnerability to groundwater diversion from the rock, and hence its sensitivity, may be lower if a low-permeability regolith such as glacial clay is present below a rich fen and thereby acts as a barrier to reduce or even prevent groundwater-table drawdown.

As an input to the sensitivity classifications at Forsmark, detailed water-flow modeling was performed to gain further insight into the effects of the groundwater diversion on water-supply mechanisms and water balances for wetland and forest objects in Forsmark (Mårtensson et al. 2010). Moreover, soil probing was performed to investigate horizontal and vertical distributions of regolith layers at delineated nature objects, and monitoring wells were installed in a number of wetland objects to enable long-term studies of groundwater and surface-water levels (Werner et al. 2010). These investigations provided object-specific data for development of local conceptual models, which supplemented regional-scale conceptual models of regolith distribution and near-surface hydrology at Forsmark (Lindborg 2008).

The investigations mentioned above and other reported investigations of ecohydrological prerequisites for different types of water-dependent or water-favored habitats in Sweden, e.g., Florgård et al. (2000), were used as inputs to define the sensitivity classes for the Forsmark study, shown in Table 1. Class 1 (very high sensitivity) means that ecological consequences can occur if the long-term groundwater-table drawdown is ≤0.1 m. Examples include ponds and fens without low-permeability bottom sediments. Class 2 (high sensitivity) means that ecological consequences can occur if the groundwater-table drawdown is 0.1–0.3 m. Examples are ponds and fens that are underlain by low-permeability bottom sediments. Class 3 (sensitive) means that ecological consequences can occur if the groundwater-table drawdown is 0.3–1 m. Typical examples of this class include moist forests and shore meadows. Class 4 (less sensitive) means that ecological consequences can occur if the groundwater-table drawdown is 1–2 m. For instance, this class includes mesic forests. Class 5 (not sensitive) means that drawdown of the groundwater table cannot lead to any negative ecological consequences, which applies to, e.g., forests on dry soils.

### Assessment and Classification of Hydrological Effects

The third and, in general, computationally most demanding step of the methodology is to predict hydrological effects of groundwater diversion. Available methodologies and tools for such predictions range from relatively simple water-balance calculations and analytical solutions, to complex numerical water-flow models. At any particular site, the choice of modeling methodology and tool(s) for the assessment should be based on a site-specific conceptual hydrological model (cf. Fig. S2 in Electronic Supplementary Material), the specific objectives of the predictions, and the severity of potential ecological consequences of groundwater diversion from the rock. In the Forsmark case study, the numerical water-flow modeling tool MIKE SHE, which is fully integrated with the channel-flow code MIKE 11 (Graham and Butts 2005; Butts and Graham 2008), was used to calculate hydrological effects in terms of changes in groundwater levels, level of the groundwater table, surface-water levels, and discharges in streams. It is noted that MIKE SHE is also used as a flow modeling tool to support calculations of post-decommissioning radionuclide transport within the biosphere at Forsmark (Berglund et al. 2013a, b). The subsurface parts of the Forsmark repository were represented using the MOUSE code, originally developed for urban hydrology purposes and pipe-flow hydraulics (Graham and Butts 2005; Butts and Graham 2008). The MOUSE model, in which the repository was described as a number of pipe links with reduced pressure, was coupled with a site-specific MIKE SHE model setup that represents the integrated groundwater–surface water flow system, including groundwater in both regolith and rock (Bosson et al. 2008; Mårtensson and Gustafsson 2010; Mårtensson et al. 2010). Comprehensive, transient model calibrations were performed to support the predictive modeling, using several years of undisturbed hydrological monitoring data as well as data obtained from hydraulic borehole tests. In the base case of the predictions, transient water-flow calculations were performed using daily sea-level data and meteorological data from the year 2006. That specific year had an accumulated precipitation of 539 mm that was rather close to the long-term average of 559 mm. Moreover, simulations were performed to investigate hydrological effects of groundwater diversion for sequences of years with less or more precipitation than normal, including ranges of conceivable climate and sea-level changes up to the year 2100.

In addition to the hydrometeorological parameters mentioned above, MIKE SHE simulation cases were also executed to investigate the influence of the hydrogeological properties of regolith and rock, the number of open, water-drained tunnels in the repository, the hydraulic conductivity of grouted rock around subsurface cavities, as well as the locations of model-domain boundaries and the spatial discretization of the model domain. In order to capture influences of both temporal hydrometeorological variability and the listed parameter and model uncertainties, the simulation case chosen as input to the integrated ecohydrological assessment represents a purely hypothetical situation with a fully open, water-drained repository and a relatively permeable grouted zone (hydraulic conductivity 10^−7^ m s^−1^). Hence, this simulation case provides safety margins for the ecohydrological assessment and is likely to overestimate the groundwater-table drawdown that would occur in practice. The MIKE SHE calculation results were temporally and spatially distributed, and could hence be used to assess hydrological effects for individual nature objects. The classes shown in Table 1 were used to categorize effects, using groundwater-table drawdown as the classification parameter. These classes were based on the model-calculated annual average groundwater-table drawdown for the type year of 2006, using an average drawdown of 0.1 m to define the boundary of the influence area.

### Integrated Ecohydrological Assessment

The fourth and last step of the ERA methodology is to use the combined results of the previously described steps to assess and classify consequences for individual nature objects and their flora and fauna. Using consequence classes clearly facilitates prioritization and development of protection measures, mitigation measures and compensation measures for nature objects and/or species. There is no standard system in Sweden for classification of ecological consequences, and such classifications are by necessity based on a mixture of quantitative and qualitative information (e.g., Silvert 1997).

The classification system used in this study (Table 1) takes into account consequences of reduced availability of groundwater and/or surface water, nature values, and the ecological status of individual objects and flora and fauna species. Class 1 (very large consequences) means extinction of ecological core values for a nature object of value class 1, or extinction of core values of an assigned Natura 2000 area. Class 2 (large consequences) involves, for instance, substantial ecological changes of a nature object of value class 2. Class 3 (noticeable consequences) involves, for instance, extinction of the nature values of a nature object of value class 3. Class 4 (small consequences) involves, for instance, extinction of the nature values of a nature object of value class 4. Class 5 means that the consequences are very small, whereas class 6 implies that there are no ecological consequences.

## Results

### Delineation and Classification of Nature Objects

**Fig. 1:**
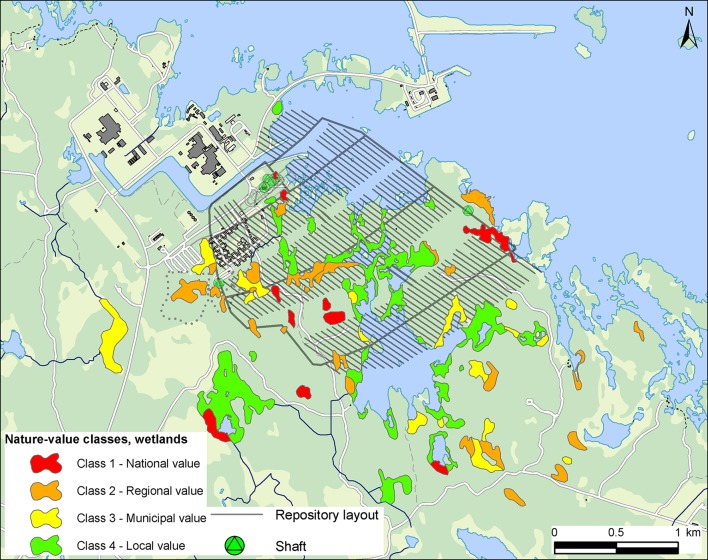
Overview map showing locations and nature-value classifications of delineated wetland objects at Forsmark

In total, 134 water-dependent or water-favored nature objects were geographically delineated and classified at the Forsmark site; 79 wetland objects, 49 forest objects, and six lakes. As shown in Fig. 1, ten wetland objects were classified as class 1 (national value), 26 as class 2 (regional value) and the remaining 43 objects were classified as class 3 or 4 (municipal or local value). One forest object was classified as class 1, whereas 23 forest objects were classified as class 2. The nature objects with the highest nature values include rich fens, lime-rich ponds, and coniferous forests located on lime-rich soils. Many of these objects host red-listed species, such as orchids and lime-associated fungi in forests, but also other delineated wetland objects contain rare species.

Soil probing showed that the bottom regolith in many wetlands consists completely or partially of low-permeability glacial clay. On the other hand, most of the delineated forest objects are located in areas that do not contain low-permeability regolith. Hence, forest objects do not have any barrier that reduces or prevents drawdown of the groundwater table in the case of groundwater-level drawdown in the underlying rock.

### Assessment of Hydrological Effects

Hydraulic borehole tests, performed as part of the site investigations at Forsmark, show that groundwater diversion from the rock has potential to yield groundwater-level drawdown in sheet joints with large horizontal extents (Follin 2008). In accord with this conceptual model (Fig. S2, in Electronic Supplementary Material), MIKE SHE calculations show that diversion of groundwater during construction and operation of the deep-rock repository has potential to yield groundwater-level drawdown in the rock across large areas (Mårtensson and Gustafsson 2010).

Modeling results also show that drawdown of the groundwater table will primarily occur in areas with permeable, steep fracture zones that are in contact with the overlying regolith, and that changes in lake-water levels and stream discharges will be small. Hence, the magnitude of the drawdown and the size of its influence area is a function of depth in the regolith and the underlying rock. Moreover, by definition drawdown is the time-varying difference between affected and unaffected conditions. By this definition, drawdown is time-dependent in a transient water-flow system, i.e., in a system influenced by temporally variable hydrometeorological conditions in terms of precipitation, air temperature and other factors influencing flow components of the hydrological cycle. As part of the Forsmark case study, drawdown maps for different depths in regolith and rock were produced as monthly or annually averages, serving as input to different types of subsequent analyses.

**Fig. 2:**
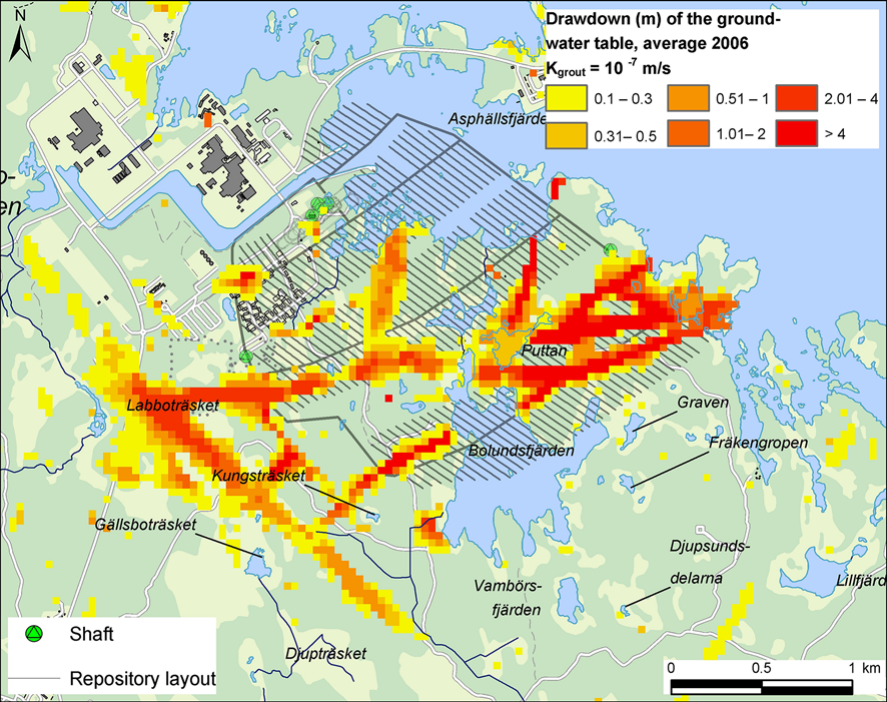
Model-calculated annual average drawdown of the groundwater table at Forsmark. *K*_grout_ denotes the hydraulic conductivity of the grouted zone

Using sea-level data and meteorological data for the type year of 2006 (the hydrometeorological base case), the purely hypothetical MIKE SHE simulation case that represents a fully open, water-drained repository and a permeable grouted zone yields an influence area for the groundwater-table drawdown, i.e., annual average drawdown of 0.1 m or larger, which is 2.5 km^2^ in size (Fig. 2); this area is smaller than the areal extent of 3–4 km^2^ of the subsurface part of the repository. As mentioned above, this simulation case provides safety margins and predicts the theoretical upper limit of the drawdown.

### Integrated Assessment of Ecohydrological Responses

**Fig. 3:**
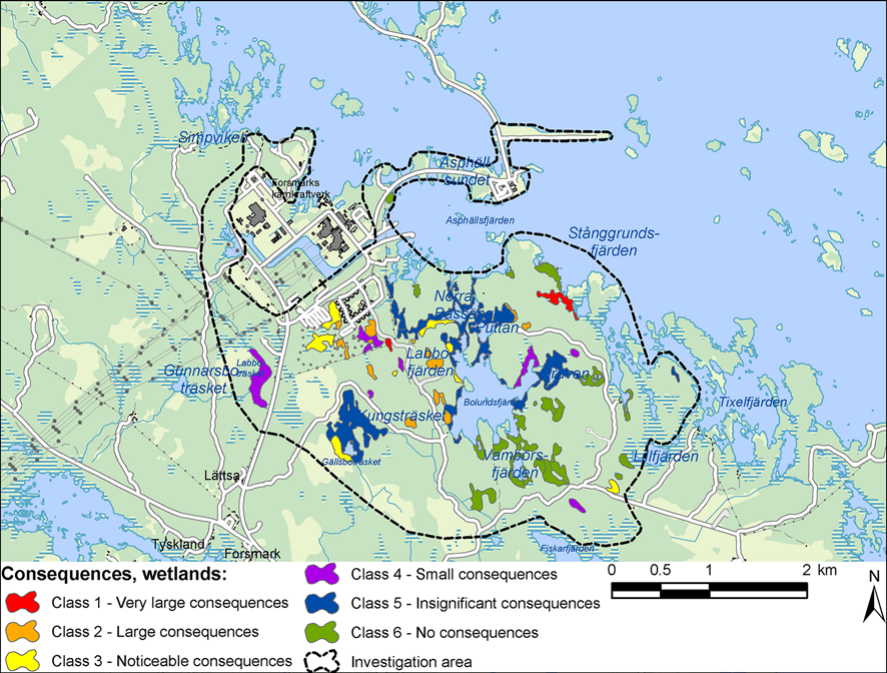
Classification of ecological consequences for wetland objects at Forsmark, without mitigation measures in the surface system

Combining the outcomes of the previously described steps, the results of the integrated ecohydrological assessment for the Forsmark case study (Fig. 3) show that without implementing any mitigating measures in the surface system, the groundwater diversion from the deep-rock repository may lead to very large consequences (i.e., consequence class 1) for two wetland objects with national value (class 1). These two objects comprise the two Natura 2000 types of rich fens and lime-rich oligo-mesotrophic waters with benthic vegetation. Moreover, ecological consequences may be large (consequence class 2) for 15 wetland objects with national or regional value. Consequences may be very large for some of the red-listed species that exist at the Forsmark site, including pool frog and fen orchid.

Forest habitats and forest species are generally less water-dependent or water-favored compared with wetland habitats and wetland species, and the former are therefore less sensitive to changes in the level of the groundwater table. Accordingly, the results of the Forsmark assessment show that the groundwater diversion will cause ecological consequences that are noticeable (consequence class 3) or of less severity for the delineated forest objects, and it is predicted that there will be noticeable consequences for red-listed forest fungi. The assessment shows that the groundwater diversion will lead to insignificant or small ecological consequences for aquatic ecosystems, due to their relatively limited nature values and the predicted small hydrological effects on such systems. There is an assigned Natura 2000 area in the easternmost part of the investigation area. The assessment shows that the protected habitats and species in this area will not be harmed or subject to any disturbance that significantly renders difficult preservation of protected species.

## Conclusions

This paper demonstrates that ecohydrological concepts and methods are important parts of planning and permit applications for construction and operation of water-drained cavities in areas with water-dependent or water-favored nature objects. Specifically, such assessments provide useful guidelines for focusing monitoring and mitigation measures on a limited number of subareas.

Underlying classification and modeling tools are inevitably associated with different types of assumptions and uncertainties, in particular assumptions and parameter and model uncertainties related to water-flow models that collectively propagate to prediction uncertainties in the integrated ecohydrological assessment. Geometries and hydraulic properties of fracture zones in crystalline rock, and hydraulic properties of the interface between the regolith and the rock, are important factors that always should be accounted for in assessments of hydrological effects. In the Forsmark case study, temporal variability of hydrometeorological parameters (including conceivable ranges of climate and sea-level changes) and uncertainties related to, e.g., the geometries and the hydraulic properties of fracture zones in the rock have been handled by comprehensive, transient model calibrations, and sensitivity analyses. The simulation case used in the Forsmark assessment captures conceivable ranges of temporal hydrometeorological variability, various parameter and model uncertainties, and it predicts the theoretical upper limit of the drawdown.

As part of preparations for repository construction and operation in Forsmark, the assessments presented in this paper are used as one of the inputs to further develop the hydrological and ecological monitoring programs, including monitoring of reference objects at other, similar sites. The EIA document that accompanies the deep-repository permit application according to the Swedish Environmental Code proposes artificial water supply to substantially reduce potential negative consequences for wetlands, i.e., habitats for rare and protected species such as the pool frog and fen orchid.

It is concluded that further research is needed, based on observed ecological responses in water-dependent or water-favored habitats to observed changes in hydrological conditions. This research requires input from systematic ecological and hydrological monitoring as part of infrastructure projects, in areas containing such habitats, which include construction and operation of water-drained cavities in rock below the groundwater table.

## Electronic supplementary material

Below is the link to the electronic supplementary material.

Supplementary material 1 (PDF 199 kb)
