# Supplementary material for: Ecohydrological Responses to Diversion of Groundwater: Case Study of a Deep-Rock Repository for Spent Nuclear Fuel in Sweden
Source: Ambio. 2013 Apr 26;42(4):517–26. doi: 10.1007/s13280-013-0404-8 (PMC3636376; doi:10.1007/s13280-013-0404-8)
Supplement: Supplementary file 1 — Supplementary material 1 (PDF 199 kb) [file 13280_2013_404_MOESM1_ESM.pdf]

Electronic Supplementary Material

Ecohydrological Responses to Diversion of Groundwater: Case Study of a Deep-Rock Repository for Spent Nuclear Fuel in Sweden

Kent Werner, Per Collinder, Sten Berglund, Erik Mårtensson

THE REPOSITORY AND THE SITE IN PICTURES

A Swedish deep-rock repository for spent nuclear fuel is planned, and Forsmark in central Sweden has been selected as the site for this.

The location of Forsmark is shown in an overview map in Kautsky et al. (2013, their Fig. 3).

Below, Figure S1 indicates the layout of the repository, while Figure S2 shows the conceptual model of pertinent hydrological conditions.

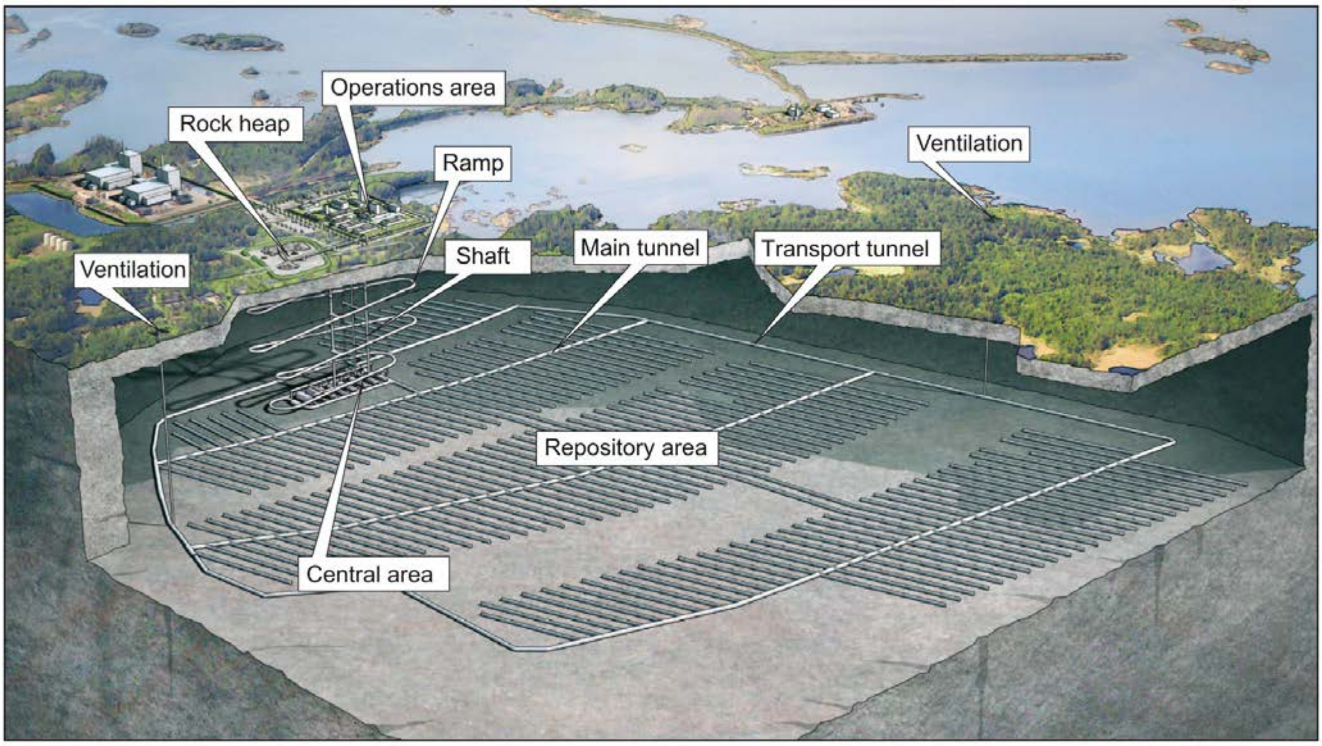

Fig. S1 Layout of the planned deep-rock repository for spent nuclear fuel at Forsmark, central Sweden

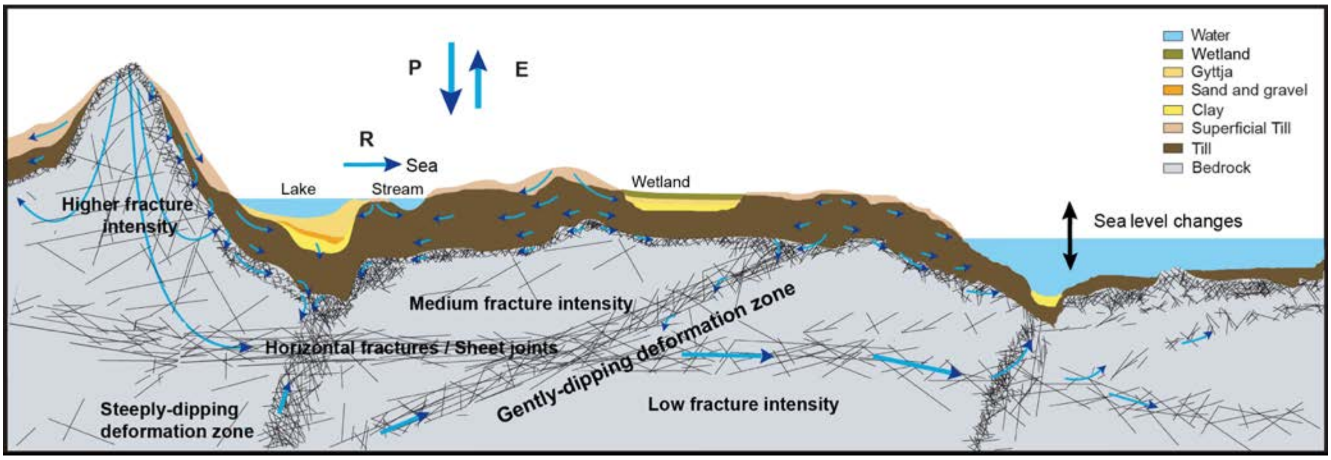

Fig. S2 Illustration of the conceptual model of hydrological conditions in the surface system and in the rock at Forsmark, down to a depth of 100–150 m (cf. Follin 2008). Note that the vertical scale is exaggerated compared with the horizontal scale. P is precipitation, E is evapotranspiration and R is runoff

REFERENCES

Follin, S. 2008. Bedrock hydrogeology Forsmark – Site descriptive modelling, SDM-Site Forsmark. Svensk Kärnbränslehantering AB, SKB R-08-95, Stockholm, Sweden, Report, 163 pp.

Kautsky, U., T. Lindborg, and J. Valentin. 2013. Humans and ecosystems over the coming millennia – overview of a biosphere assessment of radioactive waste disposal in Sweden. *AMBIO*. doi: 1007/s13280-013-0405-7
